# Effects of AMF inoculation on the eco-physiological characteristics of *Imperata cylindrica* under differing soil nitrogen conditions

**DOI:** 10.3389/fpls.2023.1134995

**Published:** 2023-06-02

**Authors:** Tong Jia, Yue Zhang, Yushan Yao, Yu Wang, Xueli Liang, Mengyao Zheng, Lijuan Zhao, Baofeng Chai

**Affiliations:** ^1^ Shanxi Laboratory for Yellow River, Shanxi Key Laboratory of Ecological Restoration on Loess Plateau, Institute of Loess Plateau, Shanxi University, Taiyuan, China; ^2^ School of Chemistry, Xi’an Jiaotong University, Xi’an, China

**Keywords:** arbuscular mycorrhizal fungi, inoculation, eco-physiological characteristics, membership functions, copper tailings areas

## Abstract

Arbuscular mycorrhizal fungi (AMF) play a key role in terrestrial ecosystems, while the ecological restoration application of AMF in mining areas has been progressively gaining attention. This study simulated a low nitrogen (N) environment in copper tailings mining soil to explore inoculative effects of four AMF species on the eco-physiological characteristics of *Imperata cylindrica*, and provided plant-microbial symbiote with excellent resistance to copper tailings. Results show that N, soil type, AMF species, and associated interactions significantly affected ammonium ( ${\text{NH}}_{4}^{\;+}$ ), nitrate nitrogen ( ${\text{NO}}_{3}^{\;-}$ ), and total nitrogen (TN) content and photosynthetic characteristics of *I. cylindrica*. Additionally, interactions between soil type and AMF species significantly affected the biomass, plant height, and tiller number of *I. cylindrica*. *Rhizophagus irregularis* and *Glomus claroideun* significantly increased TN and ${\text{NH}}_{4}^{\;+}$ content in the belowground components *I. cylindrica* in non-mineralized sand. Moreover, the inoculation of these two fungi species significantly increased belowground ${\text{NH}}_{4}^{\;+}$ content in mineralized sand. The net photosynthetic rate positively correlated to aboveground total carbon (TC) and TN content under the high N and non-mineralized sand treatment. Moreover, *Glomus claroideun* and *Glomus etunicatum* inoculation significantly increased both net photosynthetic and water utilization rates, while *F. mosseae* inoculation significantly increased the transpiration rate under the low N treatment. Additionally, aboveground total sulfur (TS) content positively correlated to the intercellular carbon dioxide (CO_2_) concentration, stomatal conductance, and the transpiration rate under the low N sand treatment. Furthermore, *G. claroideun*, *G. etunicatum*, and *F. mosseae* inoculation significantly increased aboveground ${\text{NH}}_{4}^{\;+}$ and belowground TC content of *I. cylindrica*, while *G. etunicatum* significantly increased belowground ${\text{NH}}_{4}^{\;+}$ content. Average membership function values of all physiological and ecological *I. cylindrica* indexes infected with AMF species were higher compared to the control group, while corresponding values of *I. cylindrica* inoculated with *G. claroideun* were highest overall. Finally, comprehensive evaluation coefficients were highest under both the low N and high N mineralized sand treatments. This study provides information on microbial resources and plant-microbe symbionts in a copper tailings area, while aiming to improve current nutrient-poor soil conditions and ecological restoration efficiency in copper tailings areas.

## 1 Introduction

Arbuscular mycorrhizal fungi (AMF) are the most widely distributed endophytic and mycorrhiza fungal group and the key microbes which affect terrestrial ecosystems (Gao et al., 2022). Studies have shown that different AMF communities can utilize different soil spatial resources, leading to host plant resource niche differentiation (Li, 2021). AMF can also improve plant stress resistance, which effectively enhances plant resistance to disease, drought, waterlogging, salt and alkali content, heavy metals, weeds, and high and low temperatures (Rivero et al., 2018; Li et al., 2019; Wang et al., 2020; Han et al., 2022; Zhu et al., 2022). Moreover, AMF promotes the nutrient absorption and water-use efficiency of host plants, improves their photosynthetic and osmoregulatory capacity, and contributes to improvements of their antioxidant capacity and drought resistance (Estrada et al., 2013; Han et al., 2022). Furthermore, AMF can directly or indirectly improve the stress resistance of host plants in many aspects. For example, AMF can improve plant water absorption, which would otherwise be difficult for root systems to absorb through their mycelial networks, while improving the overall water and nutritional status of plants, being instrumental in their nutritional status under stress. Through means of regulating soil microecology in the rhizosphere *via* improvements in soil organic matter (SOM) and microbial levels (Kong, 2021), exogenous mycelia can promote water absorption and regulate the transmission of plant root chemical signals (Green et al., 1998). This subsequently promotes the rapid transmission of water and nutrients to aboveground plant components, reduces stomatal conductance and transpiration rates, improves the photosynthetic capacity of plants, and regulates the osmotic capacity of plants to better cope with drought (Zhu et al., 2015). Moreover, damage to the cytoplasmic membrane can be alleviated by regulating the ion balance of plant cells under stress (Cao et al., 2015).

Symbiosis between plant roots and AMF can help improve plant nitrogen (N) and phosphorus (P) absorption efficiency (Zhou et al., 2021). AMF can symbiotically secrete various enzymes (Saia et al., 2014) and organic acids (Tawaraya et al., 2006) with plants to promote availability of P and N (as well as other nutrient) in soil. Moreover, AMF can help symbionts to form mycelial networks and bridges between plants (Whitfield, 2007). The vast surface area of mycelia can also effectively improve plant and soil interactions and promote root activity (Balogh-Brunstad et al., 2008). Mycelial bridges can directly transfer N that will subsequently be directly absorbed into the host root system. This can also affect N redistribution (Govindarajulu et al., 2005). Additionally, AMF can alter species composition and productivity under N application practices while increasing the relative abundance and aboveground biomass of plants (Zhang et al., 2016).

The role that AMF play is important for host plant photosynthetic processes (Xu, 2021). Studies have shown that AMF inoculation can effectively improve the photosynthetic capacity and carbon (C) assimilation efficiency of plants under drought stress (Metwally et al., 2019; Ye et al., 2022). Additionally, AMF can significantly increase the net photosynthetic rate of host plants, increase dry matter accumulation in plants, and enhanced plant drought resistance. Currently, it remains unclear how AMF affect photosynthetic plant processes (Xu, 2017). According to Huang et al. (2011), AMF mainly improves the nutrient absorption of host plants, which in turn helps promote the accumulation of sufficient amounts of N and P for effective photosynthesis. Additionally, Ludwig-Miiller (2010) reported that AMF inoculation may affect hormone (i.e., abscisic acid [ABA]) levels of host plants that regulate stomatal conductance, thus impacting photosynthetic efficiency (Ludwig-Miiller, 2010). Additionally, AMF species differ regarding their effect on eco-physiological host characteristics. One study found that *Rhizoglomus aggregatum*, *Glomus etunicatum*, *Glomus claroideun*, and *Funneliformis constrictus* can improve plant growth and photosynthesis (Wang et al., 2022). Among these, *R. aggregatum* plays a dominant role in promoting seedling height and *G. etunicatum* and *G. claroideun* play a dominant role in promoting root regeneration. Moreover, *R. aggregatum*, *G. etunicatum*, and *G. claroideun* can maximize the net photosynthetic rates of plants. On the other hand, *F. mosseae* can effectively alleviate a decline in the photosynthetic capacity of host plants under stress conditions (Wang et al., 2022).

Technological-based AMF approaches used in the ecological restoration of mining areas have gradually been gaining attention in recent years due to their low cost and high efficiency (Bi and Xie, 2021). For example, AMF can be used to increase vegetation survival rates while improving land reclamation efficiency (Druille et al., 2013; Hao et al., 2014). AMF not only have a positive effect on plant nutrient absorption and enzyme activities, but also can enhance the stability of soil aggregates, improve soil permeability and water retention, and boost overall soil quality (Yang et al., 2016; Choi et al., 2018). The Zhongtiao Mountains copper mining region, Shanxi Province, is North China’s largest, producing 7 million tons of copper annually. It is the largest non-coal underground mining area in China. This mining region produces vast amounts of copper tailings, resulting in severe pollution and damage to the local ecological environment. Previous studies have reported that nutrient levels are low in copper tailings ore. *Imperata cylindrica* is the dominant grass species in this region, and may form a symbiotic relationship with AMF during phytoremediation (Jia et al., 2022). Based on this hypothesis, we simulated the low N conditions of this copper tailings region to explore how four different AMF inoculation species types will affect the eco-physiological characteristics of *I. cylindrica*. For this study, we screened out plant-microbial symbiont strains to improve resistance in copper tailings areas, to enhance the status quo of nutrient scarcity, and to increase the efficiency of ecological restoration in copper tailings areas.

## 2 Materials and methods

### 2.1 Experimental material

The plant selected for this study (*I. cylindrica*) is a perennial herbaceous cogon grass species with a glabrous, erect stem that grows up to 80 cm tall. Prior to the study, *I. cylindrica* seeds were soaked in 10% hydrogen peroxide (H_2_O_2_) for 10 min before being rinsed several times with sterile water to kill miscellaneous bacteria on the seed surface (Zhang et al., 2015). The seeds of *I. cylindrica* were provide by Clover (Beijing) Ecological Technology Co., Ltd. Four AMF species were used in this study: *Glomus claroideun* (GC), *Glomus etunicatum* (GE), *Rhizophagus irregularis* (RI), and *Funneliformis mosseae* (FM). These AMF species originally provided by the Institute of Plant Nutrition and Resources, Beijing Academy of Agriculture and Forestry Sciences. Sorghum was used for strain propagation under greenhouse conditions. The soil types used for this study were typical river sand (i.e., here referred to as non-mineralized sand) and sand obtained from the copper tailings area (i.e., here referred to as mineralized sand). Impurities such as large stones and leaf detritus were removed using a 2 mm sieve and then autoclaved to eliminate any mycorrhizal fungi (or other microbe) influence in the soil samples.

### 2.2 Experiment design

A 2×5×2 completely randomized three-factor block design was used for the experiment. The first factor was the soil matrix itself: sand from the copper tailings area (i.e., mineralized sand) and sand obtained from a typical river system (i.e., non-mineralized sand). The second factor was the four AMF inocula (i.e., GC, GE, RI, FM), including a control where no inoculant was used. The third factor was the nutrient treatments, namely, the low nitrogen (LN) treatment and the high nitrogen (HN) treatment. Each treatment was replicated fivefold (i.e., 100 pots in total). Urea was used for N inoculation. Compared to the LN group, the amount of inoculum in the HN group was greater by a factor of 10, which was in accordance with the lowest N content measured in copper tailings dam (Xin et al., 2016). The nutrient solution was in the form of a Hoagland solution.

The sterilized matrix was weighed in a plastic pot (21 cm × 12 cm) filled to two-thirds of the way. The AMF inoculant (100 g) was added to the AMF treatment and then spread onto a sterilized matrix. The same amount of sterilized inoculant was added to the treatment where no inoculant was added (i.e., the control) before being covered with the sterilized matrix (2 cm). In total, 30 *I. cylindrica* seeds were sowed in each pot. After one month growth, the *I. cylindrica* seedlings were thinned to 15 in each pot. We randomly altered the position of each pot every two weeks under a three-month planting cycle in greenhouse, which was set to a temperature of 20°C at night, 25°C during the day and 50% moisture under natural light.

### 2.3 Characteristics of *I. cylindrica* plant growth and infection

Plant height and tiller number were measured at harvest time. Plant specimens were first oven-dried at 105°C for 30 min, and then further oven-dried at 65°C to a constant weight for biomass determination. Additionally, *I. cylindrica* roots were washed with clean water, immersed in a 10% potassium hydroxide (KOH) solution, and treated in a 90°C water bath for 1 h. Root samples were treated in a hydrochloric acid (HCl) solution for 3–5 min after allowing to cool and then washed with distilled water to remove pigments. Roots were sectioned into approximately 1 cm segments, stained with 0.05% Aniline Blue WS, treated in a 90°C water bath for 30 min, washed with clean water, and finally placed under a microscope for observation, where plant mycorrhizal infection rates were calculated (Liu et al., 2015). Equation (1) was used to calculate the mycorrhizal infection rate (MIR):


(1) MIR=NS/TNS×100

MIR denotes the root mycorrhizal infection rate (%); NS denotes the number of mycorrhizal segments; TNS denotes the total number of root segments.

Spore density was measured through the wet sieving and sucrose gradient centrifugation procedures and was expressed as the number of AMF spores isolated from 100 g of air-dried soil (Ren, 2017).

### 2.4 Aboveground and belowground nutrient measurements

Oven-dried plant samples were ground using a ball mill. Following this, total carbon (STC), total nitrogen (STN), and total sulfur (STS) content within aboveground plant components and total carbon (RTC), total nitrogen (RTN), and total sulfur (RTS) content in belowground plant components were measured using an elemental analyzer (vario MACRO cube, Germany). Ammonium ( ${\text{NH}}_{4}^{\;+}$ ) and nitrate nitrogen ( ${\text{NO}}_{3}^{\;-}$ ) were measured using an automated discontinuous chemical analyzer (DeChem-Tech, CleverChem380, Germany).

### 2.5 Photosynthetic pigment measurements

To measure photosynthetic pigments, we weighed leaf material (0.5 g) before soaking it in a 20 mL mixed solution, with a 1:1 acetone to ethanol ratio. Absorbance was measured at 663 nm, 645 nm, 440 nm, 644 nm, and 662 nm using a microplate reader after seven days under darkened conditions. Following this, the photosynthetic pigment content was calculated using equations (2), (3), (4), and (5):


(2) $$\text{Chlorophyll }a\text{ content}\left(\text{mg}/\text{g}\right)=\left(12.7A_{663nm}-2.69A_{645nm}\right)*V/\left(1000w\right)$$

(3) $$\text{Chlorophyll }b\text{ content}\left(\text{mg}/\text{g}\right)=\left(22.9A_{645nm}-4.68A_{663nm}\right)\times V/\left(1000w\right)$$

(4) $$\text{Total chlorophyll content}\left(\text{mg}/\text{g}\right)=\left(20.3A_{645_{nm}}-8.03A_{663_{nm}}\right)\times V/\left(1000w\right)$$

(5) $$\text{Carotenoid content}\left(\text{mg}/\text{g}\right)=\left(4.7A_{440_{nm}}-5.48A_{644_{nm}}-1.38A_{662_{nm}}\right)\times V/\left(1000w\right)$$

where V represents the volume of the ethanol and acetone mixed solution, and w represents the weight of the leaf material.

### 2.6 Photosynthetic characteristics of *I. cylindrica*

Photosynthetic characteristics were measured using a photosynthetic apparatus (i.e., the CIRAS-3 Portable Photosynthesis System) on a sunny day eight weeks after the plant culture was first established. Light intensity was set at 1200 μmol/(m2·s), and the temperature was set at 25°C. The first fully expanded new leaves were selected for determination. Five replicates of each treatment were made. The photosynthetic indexes used were the net photosynthetic rate (Pn), the intercellular CO_2_ concentration (Ci), stomatal conductance (Gs), water-use efficiency (WUE), water vapor pressure deficit (VPD), and the transpiration rate (Tr).

### 2.7 Statistical analysis

SPSS.25 was used for statistical analysis. Duncan’s multiple range test was used as a *post hoc* test to determine differences between the different bacterial treatments. Origin 2021 was used to visualize statistical results. Equation (6) was used to calculate the membership functions (Jin et al., 2018):


(6) $$\text{Membership value}=(x-x_{\min})/(x_{\max}-x_{\min})$$

where X is the measured value; X_max_ is the maximum value; X_min_ is the minimum value. Membership function values were collected and their average value was calculated for this comprehensive evaluation.

## 3 Results

### 3.1 Plant growth and AMF infection

**Figure 1:**
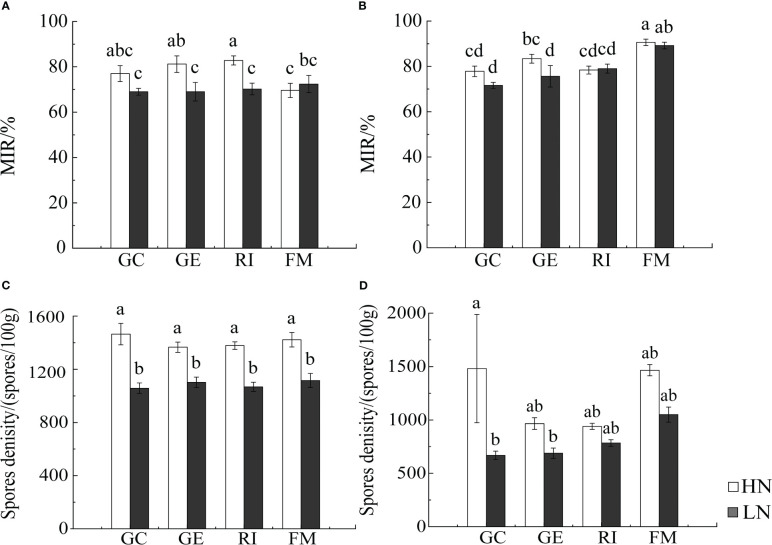
Infection rate of *I. cylindrica* and spore density in non-mineralized sand **(A, C)** and mineralized sand **(B, D)**. MIR is mycorrhizal infection rate. Different lowercase letters representing AMF species and different nitrogen had significant effects on infection characteristics and spore density (*P*<0.05).

**Figure 2:**
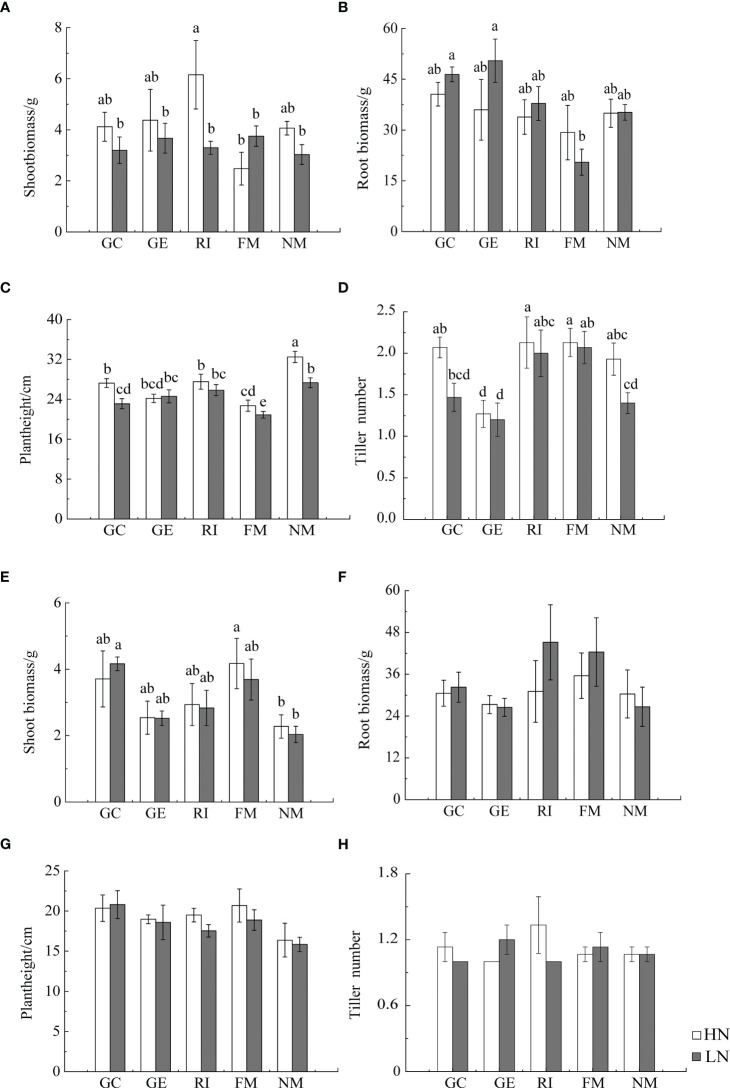
Effects of AMF inoculation on growth characteristics of *I. cylindrica* in non-mineralized sand **(A–D)** and mineralized sand **(E–H)**. Different lowercase letters represent significant differences (*P*< 0.05).

Results from Multi-Way ANOVA showed that belowground biomass, aboveground biomass, plant height, and tiller number of *I. cylindrica* were significantly affected by interactions between soil type and AMF infection type. Moreover, N, soil type, and AMF infection type all significantly affected *I. cylindrica* root infection rates and tiller numbers (*P*< 0.05, **Table 1**). The mycorrhizal infection rate of GE and RI inoculant in non-mineralized sand was significantly higher compared to the LN and HN treatments. The mycorrhizal infection rate of the GE inoculant under the HN treatment was significantly higher compared to the LN treatment (*P*< 0.05, **Figure 1**). Spore density of the four different AMF species was significantly higher under the non-mineralized sand and the HN treatment compared to the LN treatment (*P*< 0.05, **Figure 1**). After GC inoculation, spore density was significantly higher under the mineralized sand and the HN treatment compared to the LN treatment (*P*< 0.05, **Figure 1**). AMF infection promoted the shoot biomass in mineralized sand (*P*< 0.05, **Figure 2**).

**Table 1:** Multi-way ANOVA of different nitrogen and AMF infection on plant growth and the infection characteristics of *I. cylindrica*.

	Root biomass		Shoot biomass		Plant height		Tiller number		Mycorrhizal infection		Spores density	
	g		g		cm				%		spores/100g	
	*F*	*P*	*F*	*P*	*F*	*P*	*F*	*P*	*F*	*P*	*F*	*P*
Nitrogen	1.123	0.292	2.704	0.104	8.067	**0.006**	4.760	**0.032**	16.105	**<0.001**	6.770	**0.012**
Soil Type	1.341	0.250	6.634	**0.012**	135.295	**<0.001**	82.645	**<0.001**	23.747	**<0.001**	0.376	0.542
AMF	0.563	0.690	1.615	0.179	2.114	0.087	5.103	**0.001**	3.752	**0.015**	0.968	0.414
Nitrogen × Soil Type	0.005	0.942	1.874	0.175	1.942	0.167	2.678	0.106	1.854	0.178	0.823	0.368
Nitrogen × AMF	0.432	0.785	1.177	0.327	0.621	0.649	1.269	0.289	2.630	0.058	1.191	0.320
Soil Type × AMF	2.867	**0.028**	3.569	**0.010**	11.598	**<0.001**	4.236	**0.004**	8.531	**<0.001**	0.788	0.505
Nitrogen × Soil Type × AMF	0.738	0.569	1.688	0.161	1.081	0.371	0.798	0.530	1.677	0.181	0.892	0.450

AMF represents the inoculation of a single arbuscular mycorrhizal fungus, and the bold number represents a significant impact (*P*<0.05). The symbol “×” represent the interaction.

### 3.2 Effects of AMF inoculation on *I. cylindrica* characteristics

**Figure 3:**
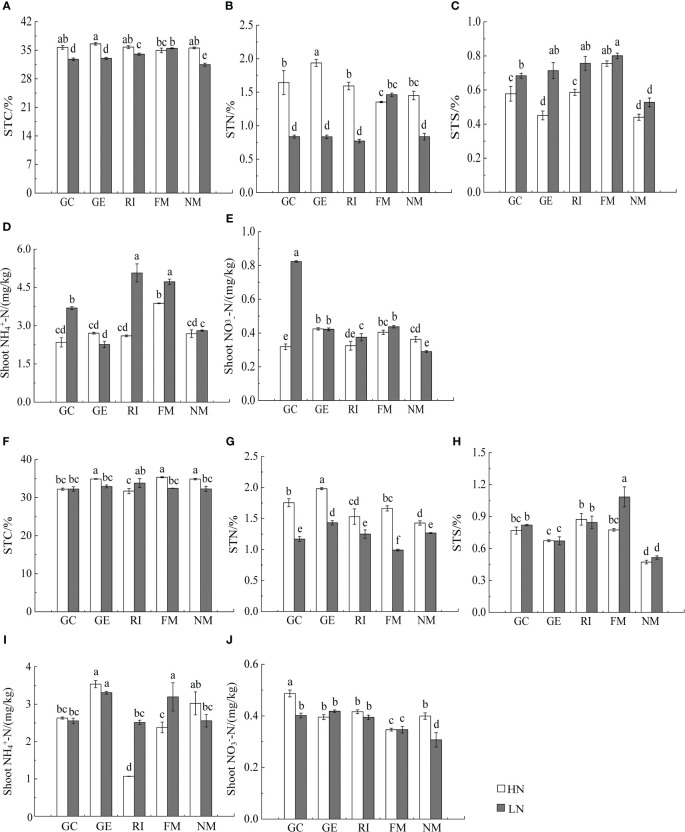
Effects of AMF inoculation on aboveground nutrient characteristics of *I. cylindric* in non-mineralized sand **(A–E)** and mineralized sand **(F–J)**. STN is shoot total nitrogen, STC is shoot total carbon, STS is shoot total sulfur. Different lowercase letters represent significant differences (*P*< 0.05).

**Figure 4:**
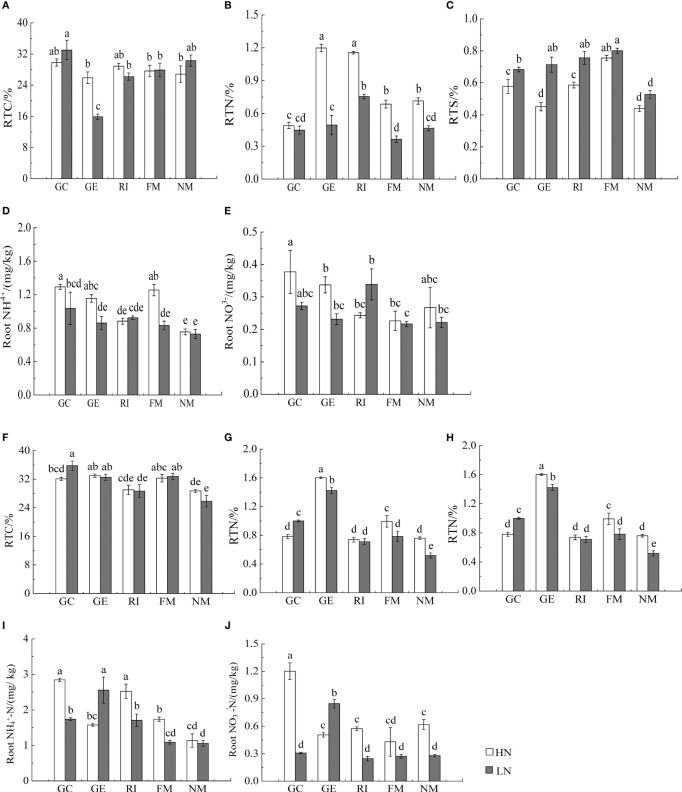
Effects of AMF inoculation on underground nutrient characteristics of *I. cylindric* in non-mineralized sand **(A–E)** and mineralized sand **(F–J)**. RTN is root total nitrogen, RTC is root total carbon, RTS is root total sulfur. Different lowercase letters represent significant differences (*P*< 0.05).

Results showed that N, soil type, AMF infection, and associated interactions significantly affected ${\text{NH}}_{4}^{\;+}$ , ${\text{NO}}_{3}^{\;-}$ , and total nitrogen (TN) content (*P*< 0.05, **Tables 2**, **3**), which also significantly affected below ground ${\text{NO}}_{3}^{\;-}$ and TN content in *I. cylindrica* (*P*< 0.05, **Table 3**). The FM inoculant under the LN treatment significantly increased aboveground total carbon (TC) content in non-mineralized sand (*P*< 0.05, **Figure 3**), while the RI inoculant significantly increased belowground TN content (*P*< 0.05, **Figure 4**). Moreover, the GE and FM inocula under the HN treatment significantly increased the TN content of belowground *I. cylindrica* components in mineralized sand (*P*< 0.05, **Figure 4**). The GE inoculant under the HN treatment significantly increased the TN content of aboveground *I. cylindrica* components in non-mineralized sand (*P*< 0.05, **Figure 3**), while the FM inoculant under the LN treatment significantly increased the corresponding TN content in aboveground *I. cylindrica* components. The GC, GE, and FM inocula under the LN treatment significantly increased the TC content in belowground *I. cylindrica* components in mineralized sand (*P*< 0.05, **Figure 4**). The GE inoculant under the HN treatment significantly increased the ${\text{NH}}_{4}^{\;+}$ content of aboveground *I. cylindrica* components in mineralized sand (*P*< 0.05). Furthermore, ${\text{NH}}_{4}^{\;+}$ content in aboveground *I. cylindrica* components under the LN treatment was significantly higher compared to the control in mineralized sand (*P*< 0.05) (**Figure 3**).

**Table 2:** Multi-way ANOVA of different nitrogen treatments and AMF infection on aboveground nutrient characteristics of *I cylindrica*.

	Shoot ${\text{NH}}_{4}^{\;+}-\text{N}$ mg/kg		Shoot ${\text{NO}}_{3}^{\;-}-\text{N}$ mg/kg		STS %		STN %		STC %	
	*F*	*P*	*F*	*P*	*F*	*P*	*F*	*P*	*F*	*P*
Nitrogen	65.427	**<0.001**	30.958	**<0.001**	40.277	**<0.001**	428.046	**<0.001**	56.421	**<0.001**
Soil Type	69.310	**<0.001**	19.566	**<0.001**	53.989	**<0.001**	33.864	**<0.001**	26.240	**<0.001**
AMF	16.280	**<0.001**	88.533	**<0.001**	57.376	**<0.001**	12.218	**<0.001**	6.120	**<0.001**
Nitrogen × Soil Type	15.646	**<0.001**	130.562	**<0.001**	3.301	0.073	17.584	**<0.001**	23.942	**<0.001**
Nitrogen × AMF	32.452	**<0.001**	62.753	**<0.001**	1.747	0.148	11.754	**<0.001**	6.506	**<0.001**
Soil Type × AMF	55.236	**<0.001**	30.309	**<0.001**	3.797	**0.007**	6.126	**<0.001**	11.772	**<0.001**
Nitrogen × Soil Type × AMF	4.447	**0.003**	89.467	**<0.001**	7.715	**<0.001**	22.965	**<0.001**	13.434	**<0.001**

STN is shoot total nitrogen; STC is shoot total carbon; STS is shoot total sulfur. The bold number represents a significant impact (*P*<0.05). The symbol “×” represent the interaction.

**Table 3:** Multi-way ANOVA of different nitrogen and AMF infection on underground nutrient characteristics of *I cylindrica*.

	Root ${\text{NH}}_{4}^{\;+}-\text{N}$ mg/kg		Root ${\text{NO}}_{3}^{\;-}-\text{N}$ mg/kg		RTN %		RTC %		RTS %	
	*F*	*P*	*F*	*P*	*F*	*P*	*F*	*P*	*F*	*P*
Nitrogen	21.673	**<0.001**	44.884	**<0.001**	137.052	**<0.001**	0.786	0.378	4.796	**0.031**
Soil Type	213.215	**<0.001**	122.038	**<0.001**	190.628	**<0.001**	41.527	**<0.001**	5.888	**0.017**
AMF	24.852	**<0.001**	16.556	**<0.001**	119.735	**<0.001**	11.971	**<0.001**	147.249	**<0.001**
Nitrogen × Soil Type	1.597	0.210	27.301	**<0.001**	47.874	**<0.001**	1.117	0.294	1.769	0.187
Nitrogen × AMF	10.610	**<0.001**	18.839	**<0.001**	21.653	**<0.001**	5.690	**<0.001**	20.818	**<0.001**
Soil Type × AMF	11.310	**<0.001**	8.074	**<0.001**	71.608	**<0.001**	13.991	**<0.001**	82.224	**<0.001**
Nitrogen × Soil Type × AMF	11.880	**<0.001**	19.188	**<0.001**	6.224	**<0.001**	4.550	**0.002**	24.364	**<0.001**

RTN is root total nitrogen; RTC is root total carbon; RTS is root total sulfur. The bold number represents a significant impact (*P*<0.05). The symbol “×” represent the interaction.

The GC inoculant in non-mineralized sand significantly increased the ${\text{NH}}_{4}^{\;+}$ content in belowground *I. cylindrica* components (*P*< 0.05). The GC and RI inocula significantly increased the ${\text{NH}}_{4}^{\;+}$ content in belowground *I. cylindrica* components in mineralized sand (*P*< 0.05). The GC inoculant under the HN treatment significantly increased the ${\text{NO}}_{3}^{-}$ content in belowground *I. cylindrica* components (*P*< 0.05, **Figure 4**), while the GE inoculant under the LN treatment significantly increased the ${\text{NH}}_{4}^{\;+}$ content in belowground *I. cylindrica* components in mineralized sand (*P*< 0.05).

### 3.3 Effects of AMF inoculation on photosynthetic pigments and photosynthetic characteristics of *I. cylindrica*

**Figure 5:**
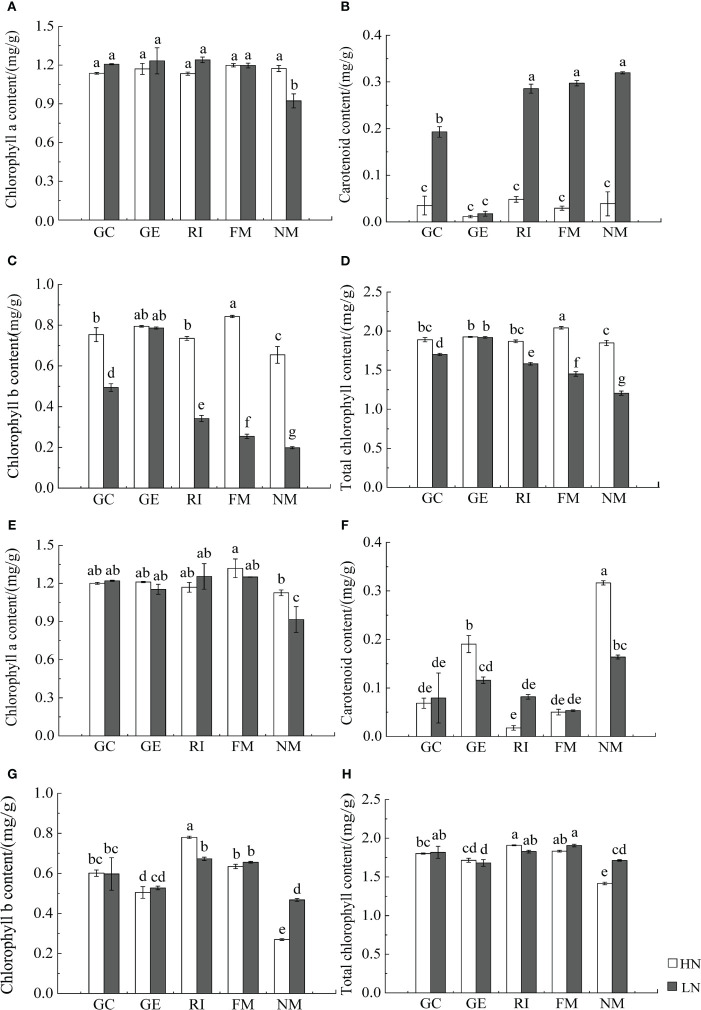
Effect of AMF inoculation on photosynthetic pigment content of *I. cylindric* in non-mineralized sand **(A–D)** and mineralized sand **(E–H)**. Different lowercase letters represent significant differences (*P*< 0.05).

AMF inoculation and associated interactions with N significantly affected photosynthetic pigments of *I. cylindrica* (*P*< 0.05, **Table 4**). AMF inoculation significantly increased the chlorophyll *a* content of *I. cylindrica* under the LN treatment in both mineralized and non-mineralized sand (*P*< 0.05) (**Figure 5**).

**Table 4:** Multi-way ANOVA of different nitrogen treatments and AMF infection on the photosynthetic pigment content of *I. cylindrica*.

	Chlorophyll a		Chlorophyll b		Carotenoid		Total chlorophyll	
	mg/g		mg/g		mg/g		mg/g	
	*F*	*P*	*F*	*P*	*F*	*P*	*F*	*P*
Nitrogen	1.315	0.255	208.978	**<0.001**	137.668	**<0.001**	157.383	**<0.001**
Soil Type	0.950	0.333	1.737	0.191	4.111	**0.046**	2.708	0.104
AMF	11.118	**<0.001**	71.767	**<0.001**	44.263	**<0.001**	80.982	**<0.001**
Nitrogen × Soil Type	1.048	0.309	283.759	**<0.001**	259.311	**<0.001**	295.639	**<0.001**
Nitrogen × AMF	6.914	**<0.001**	22.330	**<0.001**	22.960	**<0.001**	12.862	**<0.001**
Soil Type × AMF	0.954	0.437	51.663	**<0.001**	53.412	**<0.001**	32.170	**<0.001**
Nitrogen × Soil Type × AMF	0.378	0.824	28.710	**<0.001**	19.995	**<0.001**	58.185	**<0.001**

The bold number represents a significant impact (*P*< 0.05). The symbol “×” represent the interaction.

**Figure 6:**
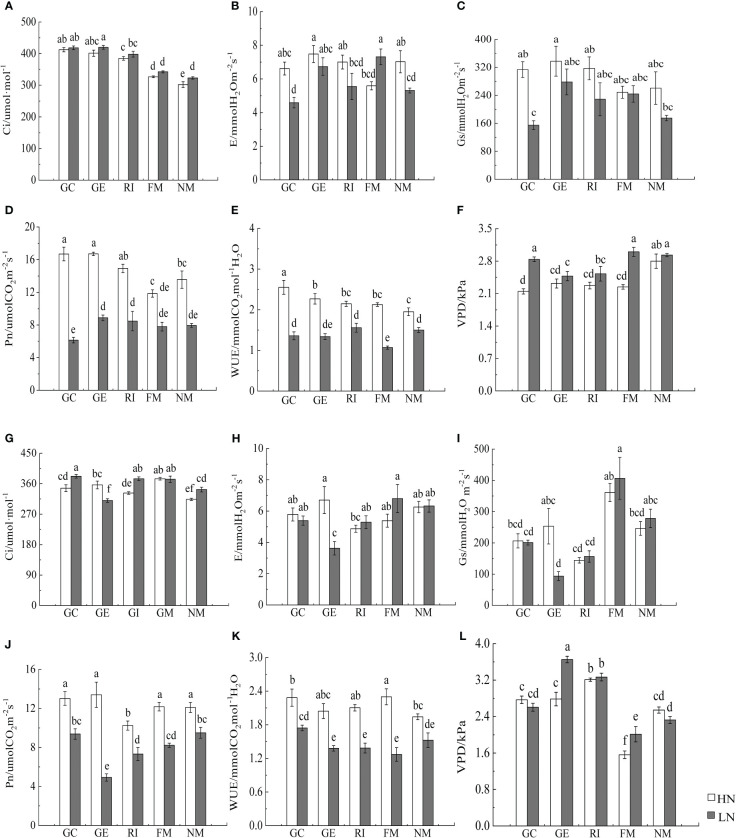
Effects of AMF inoculation on photosynthetic parameters of *I. cylindric* in non-mineralized sand **(A–F)** and mineralized sand **(G–L)**. Ci is intercellular CO_2_ concentration, Gs is stomatal conductance, VPD is vapor pressure deficit, Pn is net photosynthetic rate, Tr is evaporation rate, WUE is water use efficiency. Different lowercase letters represent significant differences (*P*< 0.05).

Furthermore, N, soil type, AMF infection, and associated interactions significantly affected the photosynthetic rate, the intercellular CO_2_ concentration, the transpiration rate, and the water vapor pressure deficit of *I. cylindrica* (*P*< 0.05, **Table 5**). AMF inoculation significantly increased the intercellular CO_2_ concentration of *I. cylindrica* in non-mineralized sand (*P*< 0.05, **Figure 6**). The GE and RI inocula under the LN treatment significantly increased the water vapor pressure deficit of *I. cylindrica* in mineralized sand, while the FM inoculant under the HN treatment significantly increased the water utilization rate of *I. cylindrica* (*P*< 0.05, **Figure 6**). The GC and GE inocula under the HN treatment significantly increased the net photosynthetic rate and the water utilization rate of *I. cylindrica* in non-mineralized sand, while the FM inoculant under the LN treatment significantly increased the transpiration rate of *I. cylindrica* (*P*< 0.05, **Figure 6**).

**Table 5:** Multi-way ANOVA of different nitrogen treatments and AMF infection on the photosynthetic parameters of *I. cylindrica*.

	Pn		WUE		Ci		Tr		VPD		Gs	
	µmol CO_2_ m^-2^·s^-1^		mmolCO_2·_mol^-1^H_2_O		µmol·mol^-1^		mmolH_2_O m^-2^·s^-1^		kPa		mmolH_2_O m^-2^·s^-1^	
	*F*	*P*	*F*	*P*	*F*	*P*	*F*	*P*	*F*	*P*	*F*	*P*
Nitrogen	19.311	**<0.001**	0.062	0.805	52.914	**<0.001**	9.316	**0.003**	7.176	**0.009**	1.311	0.256
Soil Type	2.681	**0.037**	5.142	0.001	59.827	**<0.001**	1.67	0.165	26.642	**<0.001**	3.896	0.006
AMF	374.97	**<0.001**	283.275	**<0.001**	19.003	**<0.001**	6.816	**0.011**	47.611	**<0.001**	6.464	0.013
Nitrogen × Soil Type	8.273	**<0.001**	1.414	0.237	50.287	**<0.001**	3.094	**0.021**	59.431	**<0.001**	7.596	**<0.001**
Nitrogen × AMF	19.921	**<0.001**	3.564	0.063	0.276	0.601	1.427	0.236	5.333	**0.024**	2.984	0.088
Soil Type × AMF	8.554	**<0.001**	5.188	0.001	6.305	**<0.001**	6.964	**<0.001**	7.417	**<0.001**	1.465	0.221
Nitrogen × Soil Type × AMF	5.424	0.001	2.284	0.067	8.259	**<0.001**	3.421	**0.012**	8.586	**<0.001**	1.428	0.232

Ci is Intercellular CO_2_ concentration; Gs is Stomatal conductance; VPD is Vapor pressure deficit; Pn is Net photosynthetic rate; Tr is Evaporation rate; WUE is Water use efficiency. Different lowercase letters representing AMF species and different nitrogen had significant effects on photosynthetic parameters of *I. cylindrica* in mineralized sand (*P*<0.05). The bold number represents a significant impact (*P*<0.05). The symbol “×” represent the interaction.

### 3.4 Correlation analysis between photosynthetic characteristics and physicochemical properties of *I. cylindrica*

The net photosynthetic rate positively correlated with aboveground TC and TN content (*P*< 0.05), while water-use efficiency significantly and positively correlated with belowground ${\text{NH}}_{4}^{\;+}$ content (*P*< 0.05) under the HN treatment in non-mineralized sand (**Table 6**). The transpiration rate significantly and positively correlated with aboveground TC content, aboveground TN content, and aboveground total sulfur (TS) content under the LN treatment in non-mineralized sand (*P*< 0.05) (**Table 6**).

**Table 6:** Correlation analysis between photosynthetic characteristics and physicochemical properties of *I. cylindrica* under different treatments.

Treatments			Shoot ${\text{NO}}_{3}^{\;-}-\text{N}$	Shoot ${\text{NH}}_{4}^{\;+}-\text{N}$	Root ${\text{NH}}_{4}^{\;+}-\text{N}$	Root ${\text{NO}}_{3}^{\;-}-\text{N}$	STN	STC	STS	RTN	RTC	RTS	Ci	Gs	VPD	Pn	Tr
**NMS**	**HN**	Shoot NH_4_^+^-N	0.360														
		Root NH_4_^+^-N	0.120	0.260													
		Root NO_3_^-^-N	-0.070	-0.310	0.160												
		STN	0.360	-0.501*	0.090	0.230											
		STC	0.160	-0.401*	-0.030	0.170	0.557**										
		STS	0.040	0.606**	0.448*	-0.260	-0.310	-0.501*									
		RTN	0.260	-0.120	-0.280	-0.070	0.399*	0.370	-0.290								
		RTC	-0.406*	-0.110	0.220	-0.070	-0.220	-0.200	0.210	-0.080							
		RTS	0.390	0.814**	0.549**	-0.180	-0.340	-0.350	0.730**	-0.424*	-0.080						
		Ci	-0.180	-0.479*	0.455*	0.415*	0.563**	0.320	-0.070	0.240	0.150	-0.310					
		Gs	-0.060	-0.270	0.130	0.210	0.436*	0.270	-0.180	0.160	-0.190	-0.180	0.641**				
		VPD	0.040	-0.110	-0.644**	-0.100	-0.180	-0.010	-0.415*	0.040	-0.060	-0.260	-0.713**	-0.676**			
		Pn	-0.160	-0.583**	0.180	0.260	0.548**	0.427*	-0.502*	0.130	-0.170	-0.509**	0.696**	0.612**	-0.415*		
		Tr	-0.030	-0.421*	-0.190	0.210	0.492*	0.360	-0.492*	0.250	-0.320	-0.418*	0.424*	0.902**	-0.310	0.607**	
		WUE	-0.130	-0.190	0.438*	0.050	0.090	0.110	-0.020	-0.160	0.170	-0.100	0.340	-0.290	-0.160	0.460*	-0.417*
	**LN**	Shoot NH_4_^+^-N	0.170														
		Root NH_4_^+^-N	0.298	0.055													
		Root NO_3_^-^-N	0.059	0.401*	0.073												
		STN	0.337	-0.144	-0.287	-0.052											
		STC	0.608**	0.088	0.055	0.018	0.656**										
		STS	0.562**	0.228	0.021	0.168	0.346	0.669**									
		RTN	0.252	0.174	0.518**	-0.229	-0.451*	0.003	-0.013								
		RTC	0.322	0.163	0.110	0.344	0.105	-0.067	-0.311	-0.083							
		RTS	0.285	-0.036	-0.347	0.276	0.913**	0.585**	0.393	-0.630**	0.184						
		Ci	-0.089	0.298	0.392	0.538**	-0.429*	0.032	0.293	0.277	-0.376	-0.270					
		Gs	-0.015	-0.053	-0.029	-0.321	0.195	0.326	0.385	0.129	-0.581**	0.071	0.275				
		VPD	0.118	-0.081	-0.343	0.130	0.411*	-0.005	-0.212	-0.468*	0.559**	0.472*	-0.667**	-0.759**			
		Pn	-0.048	-0.064	-0.051	-0.531**	-0.058	0.081	0.230	0.260	-0.453*	-0.211	0.028	0.811**	-0.679		
		Tr	0.034	-0.165	-0.177	-0.355	0.523**	0.457*	0.398*	-0.122	-0.475*	0.391	0.008	0.919**	-0.468	0.703**	
		WUE	-0.079	0.182	0.126	-0.134	-0.709**	-0.468*	-0.241	0.461*	0.156	-0.725**	-0.055	-0.389	-0.042	0.110	-0.602**
**MS**	**HN**	Shoot NH_4_^+^-N	-0.100														
		Root NH_4_^+^-N	0.602**	-0.489*													
		Root NO_3_^-^-N	0.756**	0.011	0.544**												
		STN	0.002	0.375	0.188	0.068											
		STC	-0.627**	0.578**	-0.731**	-0.403*	0.264										
		STS	0.183	-0.606**	0.658**	0.050	-0.029	-0.608**									
		RTN	-0.272	0.553**	-0.351	-0.316	0.636**	0.455*	-0.052								
		RTC	-0.027	0.339	0.088	0.029	0.510**	0.122	0.220	0.605**							
		RTS	-0.124	-0.189	0.008	0.141	-0.329	0.091	0.122	-0.340	0.217						
		Ci	-0.188	0.102	0.098	-0.058	0.353	0.251	0.402*	0.445*	.586**	0.285					
		Gs	-0.429*	0.384	-0.366	-0.159	0.078	0.582**	-0.197	0.209	0.229	0.355	0.664**				
		VPD	0.538**	-0.234	0.365	0.239	0.028	-0.625**	0.127	-0.086	-0.305	-0.658**	-0.617**	-0.857**			
		Pn	0.041	0.578**	-0.089	0.185	0.443*	0.263	-0.335	0.290	0.292	-0.102	0.346	0.645**	-0.338		
		Tr	0.050	0.554**	-0.259	0.125	0.229	0.312	-0.414*	0.291	0.052	-0.276	0.316	0.631**	-0.200	0.796**	
		WUE	-0.056	-0.136	0.268	0.017	0.205	-0.124	0.251	-0.041	0.339	0.341	-0.013	-0.167	-0.150	-0.011	-0.606**
	**LN**	Shoot NH_4_^+^-N	0.202														
		Root NH_4_^+^-N	0.448*	0.610**													
		Root NO_3_^-^-N	0.049	0.631**	0.416*												
		STN	0.021	0.556**	0.611**	0.275											
		STC	0.068	0.026	0.105	0.253	0.372										
		STS	-0.052	-0.109	-0.252	0.124	-0.640**	-0.096									
		RTN	0.461*	0.726**	0.843**	0.581**	0.444*	0.057	0.018								
		RTC	0.315	0.350	0.194	0.395	-0.141	-0.073	0.436*	0.593**							
		RTS	-0.202	-0.325	-0.496*	-0.200	-0.210	0.246	0.259	-0.566**	-0.438*						
		Ci	-0.481*	-0.434*	-0.710**	-0.044	-0.561**	0.093	0.574**	-0.452*	0.161	0.314					
		Gs	-0.350	-0.495*	-0.338	-0.406*	-0.462*	-0.123	0.556**	-0.377	-0.023	0.158	0.514**				
		VPD	0.213	0.713**	0.625**	0.579**	0.683**	0.210	-0.349	0.593**	-0.013	-0.043	-0.567**	-0.787**			
		Pn	-0.307	-0.598**	-0.691**	-0.461*	-0.530**	-0.207	0.028	-0.627**	0.028	0.021	0.464*	0.383	-0.688**		
		Tr	-0.389	-0.640**	-0.529**	-0.444*	-0.520**	-0.059	0.415*	-0.591**	-0.103	0.275	0.629**	0.904**	-0.819**	0.630**	
		WUE	-0.001	0.089	-0.179	-0.011	-0.043	-0.224	-0.344	0.001	0.220	-0.367	-0.158	-0.487*	0.095	0.450*	-0.383

HN is high nitrogen treatment; LN is low nitrogen treatment; NMS is non-mineralized sand; MS is mineralized sand; Ci is intercellular CO_2_ concentration; Gs is stomatal conductance; VPD is vapor pressure deficit; Pn is net photosynthetic rate; Tr is evaporation rate; WUE is water use efficiency; STN is shoot total nitrogen; STC is shoot total carbon; STS is shoot total sulfur; RTN is root total nitrogen; RTC is root total carbon; RTS is root total sulfur. Significance levels were denoted with * *P*< 0.05 and **P< 0.01.

The net photosynthetic rate significantly and positively correlated with aboveground ${\text{NH}}_{4}^{\;+}$ and TN content under the HN treatment in mineralized sand (*P*< 0.05). The transpiration rate positively correlated with aboveground ${\text{NH}}_{4}^{\;+}$ content and negatively correlated with aboveground TS content (*P*< 0.05) (**Table 6**). The net photosynthetic rate significantly and negatively correlated with ${\text{NH}}_{4}^{\;+}$ and TN content in *I. cylindrica* under the LN treatment in mineralized sand, while belowground TS content significantly and positively correlated with Ci, Gs, and Tr (*P*< 0.05, **Table 6**).

### 3.5 Membership function values and the evaluation of physiological and biochemical indexes of *I. cylindrica*

Membership function analysis is a method to comprehensively evaluate material based on multiple indexes, which avoids any bias based on a single index, evaluate each *I. cylindrica* index more comprehensively, and allow test results to be more scientifically based and reliable. This study comprehensively evaluated eco-physiological characteristics of *I. cylindrica* based on a photosynthetic index, a growth index, and aboveground and belowground nutrient content. Larger coefficient values signify better plant growth. Results showed that the average membership function value of *I. cylindrica* under AMF inoculation was higher than the control. The average membership function value of the GC inoculant was highest, while the comprehensive evaluation coefficient was highest under both the HN and LN treatments in mineralized sand (**Table 7**). The average membership function value of the RI inoculant was highest, while the average values of the membership function growth index and the photosynthetic index of the GE inoculant were highest under the LN treatment in non-mineralized sand (**Table 7**).

**Table 7:** Membership function values of each index of *I. cylindrica* under different treatments.

Treatments			Shoot ${\text{NO}}_{3}^{\;-}-\text{N}$	Shoot ${\text{NH}}_{4}^{\;+}-\text{N}$	Root ${\text{NH}}_{4}^{\;+}-\text{N}$	Root ${\text{NO}}_{3}^{\;-}-\text{N}$	Shoot biomass	Root biomass	Plant height	STN	STC	STS	RTN	RTC	RTS	Pn	WUE	Total chlorophyll	Average	Rank
**NMS**	**HN**	GC	0.000	0.000	1.000	1.000	0.447	1.000	0.464	0.497	0.451	0.944	0.000	1.000	0.281	0.996	1.000	0.217	0.605	1 (HN)
		GE	1.000	0.235	0.743	0.735	0.517	0.594	0.150	1.000	1.000	0.073	1.000	0.000	0.227	1.000	0.528	0.400	0.587	2 (HN)
		RI	0.053	0.166	0.237	0.111	1.000	0.406	0.493	0.411	0.500	1.000	0.938	0.758	0.000	0.634	0.326	0.109	0.469	4 (HN)
		FM	0.807	1.000	0.931	0.000	0.000	0.000	0.000	0.000	0.000	2.157	0.278	0.440	1.000	0.000	0.296	1.000	0.461	3 (HN)
		NM	0.417	0.222	0.000	0.271	0.432	0.505	1.000	0.167	0.367	0.000	0.317	0.236	0.163	0.350	0.000	0.000	0.296	5 (HN)
	**LN**	GC	1.000	0.510	1.000	0.456	0.235	0.865	0.346	0.096	0.331	0.572	0.213	1.000	0.442	0.000	0.594	0.694	0.511	2 (LN)
		GE	0.248	0.000	0.428	0.118	0.889	1.000	0.575	0.092	0.387	0.682	0.332	0.000	0.206	1.000	0.553	1.000	0.434	4 (LN)
		RI	0.160	1.000	0.634	1.000	0.368	0.578	0.768	0.000	0.642	0.839	1.000	0.601	0.000	0.854	1.000	0.528	0.630	1 (LN)
		FM	0.277	0.877	0.330	0.000	1.000	0.000	0.000	1.000	1.000	1.000	0.000	0.700	1.000	0.606	0.000	0.345	0.519	3 (LN)
		NM	0.000	0.194	0.000	0.039	0.000	0.491	1.000	0.095	0.000	0.000	0.257	0.841	0.134	0.657	0.885	0.000	0.306	5 (LN)
**MS**	**HN**	GC	1.000	0.632	1.000	1.000	0.756	0.395	0.923	0.590	0.135	0.738	0.050	0.790	0.596	0.880	0.955	0.782	0.696	1 (HN)
		GE	0.348	1.000	0.257	0.099	0.139	0.000	0.602	1.000	0.876	0.500	1.000	1.000	0.000	1.000	0.285	0.607	0.540	3 (HN)
		RI	0.496	0.000	0.813	0.187	0.347	0.454	0.722	0.188	0.000	1.000	0.000	0.064	0.289	0.000	0.453	1.000	0.334	4 (HN)
		FM	0.000	0.530	0.354	0.000	1.000	1.000	1.000	0.426	1.000	0.753	0.295	0.834	1.000	0.614	1.000	0.846	0.654	2 (HN)
		NM	0.376	0.791	0.000	0.245	0.000	0.368	0.000	0.000	0.867	0.000	0.025	0.000	0.498	0.589	0.000	0.000	0.251	5 (HN)
	**LN**	GC	0.849	0.051	0.456	0.101	1.000	0.310	1.000	0.400	0.000	0.536	0.532	1.000	0.000	0.974	1.000	0.607	0.547	1 (LN)
		GE	1.000	1.000	1.000	1.000	0.229	0.000	0.552	1.000	0.438	0.275	1.000	0.671	0.108	0.000	0.232	0.000	0.567	2 (LN)
		RI	0.788	0.000	0.433	0.000	0.374	1.000	0.341	0.587	1.000	0.580	0.211	0.284	1.000	0.524	0.241	0.649	0.491	3 (LN)
		FM	0.356	0.859	0.023	0.043	0.777	0.851	0.612	0.000	0.134	1.000	0.292	0.696	0.573	0.721	0.000	1.000	0.462	4 (LN)
		NM	0.000	0.054	0.000	0.053	0.000	0.010	0.000	0.623	0.015	0.000	0.000	0.000	0.430	1.000	0.536	0.138	0.181	5 (LN)

HN is high nitrogen treatment; LN is low nitrogen treatment; NMS is non-mineralized sand; MS is mineralized sand; Pn is Net photosynthetic rate; WUE is Water use efficiency; STN is shoot total nitrogen; STC is shoot total carbon; STS is shoot total sulfur; RTN is root total nitrogen; RTC is root total carbon; RTS is root total sulfur.

## 4 Discussion and conclusions

AMF play a crucial role in plant nutrient absorption and stress resistance (Zhang, 2013). Our study found that AMF species significantly affected belowground and aboveground biomass, tiller number, plant height, and mycorrhizal infection rates of *I. cylindrica*, which was consistent with a previous study (Huang, 2020). This may be because the extraradical mycelium network of AMF can penetrate areas inaccessible to plant roots, subsequently expanding the area of nutrient absorption. Additionally, the extraradical mycelium network can connect to the cortex of plants to form arbuscular structures (Ge et al., 2020), which is advantageous when water and mineral nutrients are transferred *via* plant shoots for purposes of growth and metabolism, promoting biomass accumulation (Ren et al., 2014; Zhang et al., 2018; Teng et al., 2020). The mycorrhizal infection rate can reflect symbiotic intensity between AMF and host plants (Qin, 2022), while infection rates will directly affect the ability of AMF to obtain C from host plants for its own growth requirements, thus affecting spore germination and hyphal growth (Cai, 2017). This study found that the spore density of the GC inoculant was significantly higher under the HN treatment in mineralized sand compared to the corresponding LN treatment, indicating that AMF inoculation was conducive to the germination and growth of fungal spores. Moreover, the N content of soil also affected AMF growth. This is consistent with results from a previous study (Cai, 2017). AMF infection rates will differ under different environmental factors, such as the available mineral nutrients, organic matter content, and soil pH in different regions. Studies have shown that AMF inoculation can significantly increase mycorrhizal infection rates, that excessively high N applications are not conducive to mycorrhizal infection, and that more significant root mycorrhizal infection rates will occur under LN levels. Additionally, mycorrhizal infections will vary among different plant species and different N application levels (Wang, 2012).

Being one of the three essential elements limiting plant growth and development, N is a key chemical element of plant organic matter (Cai, 2017), while its availability will be affected by soil type, N form type, etc. (Liu et al., 2019). AMF species not only absorb ${\text{NH}}_{4}^{\;+}$ and ${\text{NO}}_{3}^{\;-}$ from the surrounding environment and transfer them to host plants (Hodge et al., 2001), they also accelerate organic matter decomposition and improve plant N absorption by secreting enzymes from extraradical hypha. This study found that N content, soil type, AMF infection type, and associated interactions significantly affected the ${\text{NH}}_{4}^{\;+}$ , ${\text{NO}}_{3}^{\;-}$ , and TN content of *I. cylindrica*. Hawkins et al. (2000) reported that ${\;}^{15}{\text{NH}}_{4}^{\;+}$ absorption (per unit weight) by FM mycelia was significantly higher than that of ${\;}^{15}{\text{NO}}_{3}^{\;-}$ , with a value greater by a factor of 15. The ${\text{NH}}_{4}^{\;+}$ absorption rate (per unit weight) by mycelia was higher compared to that of ${\text{NO}}_{3}^{\;-}$ . Similarly, in non-mineralized sand the GC inoculant significantly increased the ${\text{NH}}_{4}^{\;+}$ content in belowground components of *I. cylindrica* in this study, while the GC and RI inocula in mineralized sand also significantly increased the ${\text{NH}}_{4}^{\;+}$ content in belowground components of *I. cylindrica*. Using mineralized sand as a substrate, ${\text{NO}}_{3}^{\;-}$ content in belowground components of *I. cylindrica* significantly increased in the GC inoculant under the HN treatment, while the ${\text{NO}}_{3}^{\;-}$ content in belowground components of *I. cylindrica* significantly increased in the GE inoculant under the LN treatment. The reason behind differences in AMF absorption between these two inorganic N forms could be that ${\text{NH}}_{4}^{\;+}$ requires less energy for absorption and assimilation compared to ${\text{NO}}_{3}^{\;-}$ . The absorption process of the latter is as follows: it first reduces to NH_3_ and then enters into the GS/GOGAT pathway, requiring both energy consumption and reductase participation (Wu and Ca, 2022). However, ${\text{NH}}_{4}^{\;+}$ can directly enter the GS/GOGAT pathway under conditions of low energy consumption. For ${\text{NO}}_{3}^{\;-}$ , *via* the plant root diffusion process (i.e., where it is absorbed [Sun et al., 2005]), absorption is more difficult due to mycorrhizal associations. This is because of its high mobility. On the other hand, ${\text{NH}}_{4}^{\;+}$ mobility is less robust, forming in the soil within the ${\text{NH}}_{4}^{\;+}$ enrichment region (Smith, 2010), making it easier for roots to absorb ${\text{NH}}_{4}^{\;+}$ outside the hyphae.

Photosynthesis is the fundamental means by which plants synthesize organic matter and obtain energy (Zhu et al., 2010). In this study, we found that N, soil type, AMF infection, and associated interactions significantly affected the net photosynthetic rate, the intercellular CO_2_ concentration, the transpiration rate, and the water vapor pressure deficit of *I. cylindrica*. Studies have found that AMF inoculation can also significantly increase chlorophyll content in plant leaves (Sannazzaro et al., 2006; Sheng et al., 2008; Zhu et al., 2010; Liu et al., 2011). Results from this study showed that AMF inoculation under the LN treatment significantly increased the chlorophyll *a* content of *I. cylindrica*. This may be because AMF inoculation helps *I. cylindrica* to obtain the water and nutrients necessary for metabolic photosynthetic processes to take place in belowground components, subsequently promoting chlorophyll synthesis and enhancing the photosynthetic capacity of plant leaves. Additionally, the net photosynthetic rate directly reflects the assimilation capacity of leaves (per unit area), which is an important indicator in measuring the photosynthetic capacity of plants (Hu et al., 2020). Plants provide the AMF photosynthate that most benefits them, and AMF also tends to provide soil nutrients to plants that deliver the most photosynthate for their usage (Kiers et al., 2011). Studies have also shown that AMF symbiosis can promote photosynthetic rates, transpiration rates, and a means for host plants to uptake water (Gavito et al., 2019; Puschel et al., 2020), which can improve the photosynthetic capacity of plants, although still regulated by environmental conditions and available nutrient elements. Similarly, the average membership function values of each *I. cylindrica* index inoculated with AMF were higher compared to the control. For non-mineralized sand, the net photosynthetic rate of *I. cylindrica* inoculated with GC and GE under the HN treatment significantly increased, while the net photosynthetic rate positively correlated with aboveground TC and TN content. The transpiration rate of *I. cylindrica* inoculated with FM under the LN treatment increased significantly. This may be because N enhances the enzyme activities associated with the photosynthetic electron transport chain while promoting photosynthesis, and P is an important enzyme component that is necessary for plant photosynthesis and ATP synthesis. AMF inoculation promotes N and P absorption and utilization in *I. cylindrica*, subsequently promoting plant photosynthesis (Evans and Von Caemmerer, 1996; Wu and Zhao, 2010; Wang et al., 2016). Moreover, S plays a key role in the synthesis and metabolism of photosynthetic pigments and proteases (Shao, 2004). In this study, AMF inoculation significantly increased the S content in aboveground *I. cylindrica* components, and this significantly and positively correlated with Ci, Gs, and Tr in LN mineralized sand, which was beneficial to the synthesis of various plant proteins, chlorophyll and carotenoid content, and stress resistance. In conclusion, different AMF inoculation had significant effects on the eco-physiological characteristics of *I. cylindrica* under differing soil nitrogen conditions. AMF strains can improve plant physiological characteristics to varying degrees.

## Data availability statement

The original contributions presented in the study are included in the article/supplementary material. Further inquiries can be directed to the corresponding author.

## Author contributions

TJ conceived and designed the experiments. MZ, YY, and YZ performed the experiments. BC contributed new reagents. YW, XL, LZ, and TJ wrote the manuscript. All authors read and approved the manuscript. All authors contributed to the article.
